# Urban-rural difference in the determinants of dietary and energy intake patterns: A case study in West Java, Indonesia

**DOI:** 10.1371/journal.pone.0197626

**Published:** 2018-05-16

**Authors:** Satoko Kosaka, Kazuhiro Suda, Budhi Gunawan, Ardini Raksanagara, Chiho Watanabe, Masahiro Umezaki

**Affiliations:** 1 Department of Human Ecology, School of International Health, Graduate School of Medicine, the University of Tokyo, 7-3-1 Hongo, Bunkyo-ku, Tokyo, Japan; 2 Faculty of Humanities, Hokkai Gakuen University, 4-1-40 Asahi-machi, Toyohira-ku, Sapporo, Japan; 3 Institute of Ecology, Research Institute, Padjadjaran University, Jl. Sekeloa Selatan I, Bandung, Indonesia; 4 Faculty of Medicine, Padjadjaran University, Jl. Eijkman 38, Bandung, Indonesia; Institut de recherche pour le developpement, FRANCE

## Abstract

**Background:**

Few studies have explored differences in the determinants of individual dietary/energy intake patterns between urban and rural areas.

**Objective:**

To examine whether the associations between individual characteristics and dietary/energy intake patterns differ between urban and rural areas in West Java, Indonesia.

**Methods:**

A 3-day weighed food record, interviews, and anthropometric measurements were conducted in Bandung (urban area; n = 85) and Sumedang (rural area; n = 201). Total energy intake and intake from protein, fat, and carbohydrates were calculated. Food items were grouped into dietary categories based on the main ingredients to calculate their share of total energy intake. The associations between individual characteristics and dietary/energy intake were examined by fitting regression models. Models that also included education and body mass index (BMI) were fitted to adult samples only.

**Results:**

In Sumedang, the total energy intake and energy intake from carbohydrates, fat, and grain/tubers were significantly associated with age and occupation. In Bandung, energy intake from grain/tubers and vegetables/legumes was related to sex and occupation, while other indicators showed no associations. Among adults, BMI was associated with the total energy intake and educational level was associated with energy intake from vegetables/legumes (both only in Sumedang).

**Conclusions:**

The relationship between demographic and socioeconomic factors and dietary/energy intake patterns differs in rural versus urban areas in West Java. These results suggest that different strategies are needed in rural and urban areas to identify and aid populations at risk of diet-related diseases.

## Introduction

The incidence of chronic diseases is affected by dietary factors, and many public health programs have set dietary recommendations and regulations to control increases in the prevalence of chronic diseases. However, the prevalence of diet-related chronic diseases shows few signs of decreasing worldwide. On the contrary, steep increases have been observed in low- and middle-income countries, where economic development and urbanization have introduced unhealthy diets more recently than in high-income countries. Indeed, an increasing trend in the consumption of sugars, animal products, and fats has been reported in developing countries [1], and such unhealthy diets are regarded as a major direct driver of the diabetes epidemic [2]. Moreover, along with the growth of gross domestic product per capita in low- and middle-income countries, the consumption of refined sugars, refined animal fats, oils, and alcohol are expected to increase drastically [3].

Therefore, the determinants of dietary and energy intake patterns have attracted the interest of researchers, since characterizing them may provide a theoretical basis for effective interventions. Identifying the characteristics of populations at higher risk could help policymakers prioritize and improve the efficiency of programs.

Previous studies have shown that food preferences differ between the sexes and change over an individual’s lifetime. For example, women in France tend to eat more fatty/sweetened foods, such as chocolates and cakes, than do men, and their consumption of such foods decreases with age [4]. In addition, socioeconomic status also affects food intake patterns. Vegetable and fruit intake is related to income, occupation, and educational level, and individuals with higher socioeconomic status tend to consume more fruits and vegetables; however, they also tend to have unhealthier diets [5–7].

Besides these individual characteristics, the living environment may strongly influence food intake, as indicated by the remarkable gaps observed between urban and rural areas (e.g., [8]). This is likely due to the vastly different food outlets in urban areas compared with rural areas, both qualitatively and quantitatively. Previous studies have documented the effects of neighborhood food environments, including grocery stores, convenience stores, and supermarkets, on food intake [9, 10]. Different social environments can also result in different cultural values regarding food and nutrition, such as food taboos and ideal body shape, which affect individuals’ food-related behaviors. Considering these social and food environments, it seems likely that the relationships between individual characteristics and food intake patterns differ according to the level of urbanization.

Several previous studies have demonstrated effects of the level of urbanization upon household-level food consumption (e.g., [11, 12]). However, there have been few studies carried out at the level of the individual. While some researchers recognize that socioeconomic status may affect eating behaviors differently among various social and cultural settings (e.g., [6]), the majority of previous studies have assessed the effects of socioeconomic status and location on food intake separately [5]; only a few studies have examined urban-rural differences in the determinants of individual dietary/energy intake patterns.

To address these research gaps, this case study focused on whether the relationships between demographic/socioeconomic characteristics and dietary/energy intake patterns differ between urban and rural areas in West Java, Indonesia. Asian countries, including Indonesia, have been experiencing a nutrition transition. For example, a study conducted in Indonesia reported that the proportion of energy from fat had increased 2.8-fold from 1983 to 2004 [13]. In addition, the consumption of processed foods in the country has grown 7.5% annually, where such foods are high in sugar, salt, and fat [14]. Consequently, Indonesia have experienced a marked increase in the prevalence of non-communicable diseases [15, 16]. In Indonesia, non-communicable diseases are estimated to account for 71% of all deaths [17]. Since the burden of these chronic diseases is expected to grow [18], the importance of diet and nutrition will further increase as modifiable risk factors.

## Methods

A field survey was performed between August 2014 and September 2015, covering 23 weeks in total. The Ramadan period, during which Muslims fast, was excluded. This study was approved by the Research Ethics Committee of the Graduate School of Medicine and Faculty of Medicine of the University of Tokyo (Approval Number 10485-[1]). Permission from the local governments in West Java was also obtained through Padjadjaran University.

### Study areas

West Java is one of 34 provinces in Indonesia, situated in the western part of Java Island. There are several levels and categories of administrative units in the province; it is divided into 27 *kota* and *kabupaten*, including *kota* Bandung and *kabupaten* Sumedang, where the field surveys were conducted. *Kota* Bandung, the provincial capital, is located in the center of the province, and *kabupaten* Sumedang is located in the northeast. According to the 2010 census, Bandung has a population of nearly 2.4 million people within an area of 167 km^2^ (14,317 persons/km^2^), while Sumedang has a population of 1.1 million people within 1,518 km^2^ (720 persons/km^2^). As the word “*kota*” means “city”, a large portion of Bandung consists of crowded urban areas. This contrasts starkly with Sumedang, where the land is used largely for agriculture and there are very few towns. These two areas were chosen to compare their disparate levels of economic development. Since the Sundanese constitute a large majority of the population in both Bandung and Sumedang, these areas share the same fundamental culture, values, and family systems. In addition, these two areas are at similar altitudes (~700–1,000 m above sea level) and therefore have a similar climate throughout the year.

*Kota* and *kabupaten* are divided into several smaller areas, as follows: *kecamatan*, *kelurahan*/*desa*, *rukun warga* (*RW*), and *rukun tetangga* (*RT*). The field surveys were conducted in four *RTs*, two of which were from two *RWs* in Bandung, and the other two from two *RWs* in Sumedang.

### Data collection

#### Questionnaire and anthropometry

All residents in the four *RTs* were targets for the survey. First, all households were visited and members were provided with information about the study. For the households that agreed to participate (66/88 households in Bandung and 79/83 households in Sumedang), interviews were conducted using a questionnaire that included items concerning sex, birthdate, marital status, educational attainment, employment status, occupation, and income. Anthropometric data (height and weight) were collected from all participants, and identical equipment was used at all study sites for the measurements. Height was measured using a portable stadiometer to the nearest 0.1 cm, and weight was measured using a portable digital scale in units of 100 g. Participants wore light clothing and took off their caps, shoes, and socks prior to the measurements. Children aged 2 years or younger were excluded from the anthropometric data collection.

For children and adolescents (228 months or younger), nutritional status was assessed using z-scores of height for age (HAZ), weight for age (WAZ), weight for height (WHZ), and body mass index (BMI) for age (BAZ), calculated using the WHO Child Growth Standards (Anthro and Anthro Plus). If any of the HAZ, WAZ, WHZ, or BAZ scores were below −2.0, the child was categorized as being undernourished [19]. WHZ scores for those aged 61 months or older were not calculated, since a corresponding WHZ growth reference was not provided by the WHO. The nutritional status of adults was assessed using BMI.

#### Dietary survey

The second phase of the study assessed food intake. All household members aged 2 years or older were included in the dietary survey. Due to the greater burden of this phase for participants, some of the households that agreed to participate in the first phase declined or were not able to participate in this second phase. In Bandung, eight households were selected in the first *RT*, and all the households that agreed to participate were included in the second *RT* (25 households in total). In Sumedang, all 79 households that agreed to participate in the first phase were included in the second phase.

All food and beverage items that the participants consumed were recorded for 3 days (including two weekdays and one Saturday, Sunday, or holiday) using the weighed food record method. The day before the survey period, digital scales (KD-177, Tanita, Japan) were distributed to each household along with recording sheets. During the 3-day period, the participants were asked to weigh the items they ate and drank, as well as any items left over, and to write down the time, place, name, and weight of the items on the recording sheets. Leftovers, if any, were also weighed. When participants ate outside of the home, approximate portion sizes were estimated during an interview.

The method used to weigh and record the food items was explained and demonstrated to at least one adult member in each household. To reduce errors, investigators visited households to collect and check the recording sheets every day during the study period. Since there were more elderly and illiterate participants in Sumedang, we visited those households several times a day and sometimes stayed nearby to help them. Each investigator covered three to five households at a time. However, many of the subjects in Bandung worked and ate away from their homes on most days, and elderly couples were often unable to understand and follow the instructions fully. Owing to poor-quality data, their food intake information was excluded from the dietary and nutritional analysis. As a result, our study included data from 85 individuals in 19 households in Bandung and 201 individuals in 75 households in Sumedang.

To assess energy intake patterns, total energy intake and energy intake from protein, fat, and carbohydrates were calculated using Indonesian food composition tables [20–23] and the Indonesian version of the NutriSurvey software (available at http://www.nutrisurvey.de/, downloaded in September, 2014). For dishes not found in these databases, nutritional values were estimated using common recipes in the areas, which were obtained through interviews and observations with weighing. To adjust for body size, total energy intake was divided by the basal metabolic rate calculated using Henry’s [24] equations for different sex and age categories, which are based on weight and height values. Energy intake from protein, fat, and carbohydrates was converted into percentage energy intake. Hereafter, “energy intake pattern” refers to the total energy intake and energy intake from protein, fat, and carbohydrates.

For the assessment of dietary patterns, food items were grouped into dietary categories based on the main ingredients (grain/tubers, vegetables/legumes, meat/fish, eggs, fruits, dairy products, and others), and energy intake from each dietary category was calculated. Energy intake values for rice and vegetables were calculated as subcategories, separately from the grain/tubers and vegetable/legumes categories, since great individual variation was observed for these food groups in the field. “Dietary intake pattern” hereafter refers to the energy intake from each dietary category.

### Analysis

The associations between individual characteristics and dietary/energy intake patterns were examined to explore potential predictors in each area. Linear regression models were fitted individually for each of the dietary/energy intake indicators. The independent variables included two categorical variables (sex and occupation), two continuous variables (age and per capita income), and a dummy variable that represented households. Occupation was categorized into five classes; housewife, non-sedentary jobs (e.g., agriculture and manufacturing), sedentary jobs (e.g., government office workers), student, and others (which typically included those who were retired or jobless). The distinction between sedentary and non-sedentary jobs was made according to whether the workers mist sit for long periods while at work. Since education level and body mass index (BMI) were applicable as explanatory variables only to adults, separate models were fitted for the adult-only samples, in which education level attained and BMI were included.

When occupation or education was found to be significantly associated with dietary/energy intake pattern indicators, the difference between the categories was examined using Tukey’s test.

To assess the effect of the difference in sample size between Bandung and Sumedang, *post-hoc* power analysis was conducted.

For all statistical analysis, the significance level was set at 0.05.

## Results

### Sample description

Table 1 shows the participants’ basic characteristics. The mean age of the adult participants was higher in Sumedang than in Bandung. Females had a higher BMI than males in both areas, and both sexes had higher BMIs in Bandung than in Sumedang. This reflects the demographic structure of the survey areas observed during the first phase of the study. Among the participants in the first phase, the mean ages were 41.2 years for males and 43.1 years for females in Bandung, and 47.0 years for males and 47.1 years for females in Sumedang. The mean BMI values were 22.7 for males and 25.8 for females in Bandung, and 21.9 for males and 24.1 for females in Sumedang.

**Table 1:** Characteristics of the subjects by area, sex, and adult/child status.

			Bandung (19 households)								Sumedang (75 households)							
			Adults (20y-)				Children (2-19y)				Adults (20y-)				Children (2-19y)			
			Males		Females		Males		Females		Males		Females		Males		Females	
Number of individuals			28		27		14		16		72		80		25		24	
Age (year, mean (SD))			39.9	(10.9)	40.6	(14.3)	8.4	(4.4)	8.8	(5.1)	48.2	(17.6)	47.2	(16.7)	10.8	(5.1)	9.3	(4.5)
Education (n (%))																		
	Primary or lower		7	(25.0)	5	(18.5)					46	(63.9)	56	(70.0)				
	Junior high school		4	(14.3)	6	(22.2)					12	(16.7)	13	(16.3)				
	Higschool or higher		17	(60.7)	16	(59.3)					14	(19.4)	10	(12.5)				
	Unknown		0	(0.0)	0	(0.0)					0	(0.0)	1	(1.3)				
Occupation (n (%))																		
	Housewife		0	(0.0)	20	(74.1)					0	(0.0)	39	(48.8)				
	Non-sedentary		21	(75.0)	4	(14.8)					49	(65.3)	21	(26.3)				
	Sedentary		3	(10.7)	2	(7.4)					16	(21.3)	11	(13.8)				
	Student		1	(3.6)	1	(3.7)					0	(0.0)	0	(0.0)				
	Other		3	(10.7)	0	(0.0)					7	(9.3)	9	(11.3)				
Weight (kg; mean (SD))			62.8	(8.6)	58.4	(11.5)	27.5	(16.7)	27.4	(15.3)	55.6	(10.7)	52.4	(11.8)	33.8	(18.8)	31.7	(14.6)
Height (cm; mean (SD))			162.0	(7.2)	149.9	(8.3)	122.5	(27.1)	120.5	(24.4)	159.2	(7.3)	146.8	(6.3)	131.3	(26.5)	126.9	(20.9)
BMI (mean (SD))			24.0	(3.2)	26.0	(4.9)	16.5	(3.4)	17.3	(3.4)	21.8	(3.2)	24.2	(5.1)	17.9	(3.8)	18.4	(3.4)
WAZ (mean (SD))							-2.2	(0.6)	-1.1	(0.6)					-1.7	(0.8)	-0.4	(1.2)
HAZ (mean (SD))							-1.8	(0.9)	-1.7	(0.8)					-2.1	(1.0)	-1.4	(1.3)
WHZ^a^ (mean (SD))							-0.7	(0.3)	-0.5	(1.1)					0.5	(0.9)	-0.2	(0.5)
BAZ (mean (SD))							-0.5	(1.2)	-0.1	(1.1)					-0.2	(1.4)	0.3	(0.9)

BAZ: body-mass-index-for-age z-score. BMI: body mass index. EI: energy intake. HAZ: height-for-age z-score. WAZ: weight-for-age z-score. WHZ: weight-for-height z-score. aWHZ was not calculated for children aged more than 60 months.

**Fig 1:**
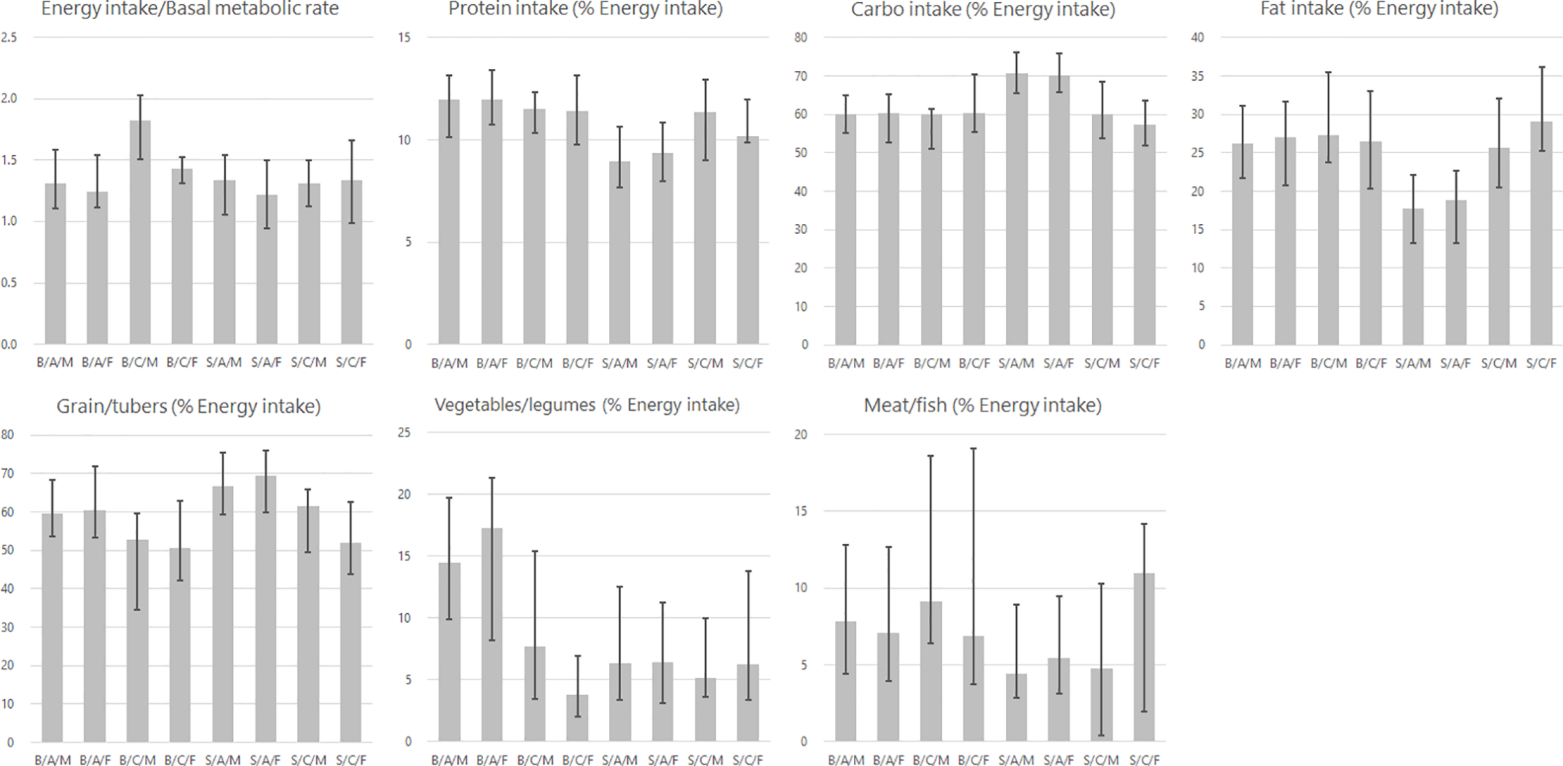
Dietary/energy intake patterns by area, adult/child status, and sex. B, Bandung; S, Sumedang; A, adult; C, child; M, male; F, female. Bars indicate median values and lines indicate 25-75th percentiles.

Fig 1 shows the dietary/energy intake patterns by area, adult/child status, and sex (see S1 Table for numerical values). Due to the non-normal distributions, they are presented as the median and 25th and 75th percentiles. The proportion of energy intake from protein and fat for adults in Sumedang was lower than those for adults in Bandung and for children in both areas, while the energy intake from carbohydrates was greater for adults in Sumedang than for the other groups. Based on the biological requirement for protein specified by the WHO, Food and Agriculture Organization of the United Nations and United Nations University ([25]; 0.66 g/kg/day), 16.7% and 40.9% of participants did not meet the criteria in Bandung and Sumedang, respectively (data not shown).

### Individual determinants of dietary/energy intake

Table 2 shows the results of fitting regression models to energy intake pattern indicators. Besides the household dummy variable, age and occupation were found to be associated, but only in Sumedang. Age was a significant determinant of energy intake after adjusting for the basal metabolic rate, and proportion of energy intake from carbohydrates and fat. Young people were more likely to have a lower overall energy intake (although the effect of age was small), lower energy intake from carbohydrates, but higher energy intake from fat. Regarding occupation, non-sedentary workers tended to obtain more energy from carbohydrates and less from fat. On Tukey’s test, a significant difference was found between other and non-sedentary categories and between other and sedentary categories, for both carbohydrate and fat intake. In Bandung, however, no significant association was found between individual characteristics and energy intake pattern indicators.

**Table 2:** Association between energy intake pattern and individual characteristics.

				Bandung		Sumedang	
*Energy intake (EI/BMR)*							
	Categorical variables			adjusted mean	p-value	adjusted mean	p-value
		Sex			0.33		0.51
			Male	1.43		1.35	
			Female	1.33		1.30	
		Occupation			0.82		0.23
			Housewife	1.37		1.33	
			Non-sedentary	1.31		1.37	
			Sedentary	1.29		1.23	
			Student	1.44		1.25	
			Other	1.48		1.45	
	Continuous variables			coefficient	p-value	coefficient	p-value
		Age		0.00	0.59	-0.01	0.01
		Per capita income*		0.09	0.52	0.14	0.07
	Dummy variable				p-value		p-value
		Household			0.01		<0.01
*Protein intake (%EI)*							
	Categorical variables			adjusted mean	p-value	adjusted mean	p-value
		Sex			0.77		0.91
			Male	11.6		10.0	
			Female	11.6		10.0	
		Occupation			0.27		0.64
			Housewife	12.3		9.9	
			Non-sedentary	11.8		9.6	
			Sedentary	12.7		9.8	
			Student	11.4		10.3	
			Other	10.4		10.3	
	Continuous variables			coefficient	p-value	coefficient	p-value
		Age		0.00	0.80	-0.02	0.07
		Per capita income*		-0.17	0.82	-0.34	0.42
	Dummy variable				p-value		p-value
		Household			<0.01		<0.01
*Carbohydrate intake (%EI)*							
	Categorical variables			adjusted mean	p-value	adjusted mean	p-value
		Sex			0.96		0.86
			Male	60.4		67.2	
			Female	60.4		67.4	
		Occupation			0.41		0.01
			Housewife	59.8		68.2	
			Non-sedentary	58.3		69.3	
			Sedentary	58.9		70.1	
			Student	60.8		65.0	
			Other	63.7		63.8	
	Continuous variables			coefficient	p-value	coefficient	p-value
		Age		0.03	0.59	0.16	<0.01
		Per capita income*		2.18	0.37	0.31	0.83
	Dummy variable				p-value		p-value
		Household			<0.01		<0.01
*Fat intake (%EI)*							
	Categorical variables			adjusted mean	p-value	adjusted mean	p-value
		Sex			0.81		0.95
			Male	26.6		21.2	
			Female	27.0		21.1	
		Occupation			0.74		0.01
			Housewife	26.9		20.4	
			Non-sedentary	28.7		19.5	
			Sedentary	26.8		18.5	
			Student	26.3		23.0	
			Other	25.3		24.5	
	Continuous variables			coefficient	p-value	coefficient	p-value
		Age		-0.04	0.50	-0.15	<0.01
		Per capita income*		-1.53	0.51	-0.18	0.88
	Dummy variable				p-value		p-value
		Household			<0.01		<0.01

*million rupiah per month. EI: energy intake (kcal). BMR: basal metabolic rate (kcal).

The associations between individual characteristics and dietary intake patterns are shown in Table 3. Dietary categories with a greater contribution to total energy intake were assessed. Energy intake from grain/tubers was significantly associated with occupation in Bandung. Sedentary workers tended to have greater energy intake from grain/tubers than the other categories of workers, although a significant difference was found only with the combination of other and non-sedentary job category combination. In Sumedang, energy intake from grain/tubers was positively associated with age; as age increased, so too did energy intake. Several factors were associated with energy intake from vegetables/legumes only in Bandung; for example, males tended to obtain more energy from vegetables/legumes. No association was found between energy intake from meat/fish and individual characteristics in either area.

**Table 3:** Association between grain/tuber, vegetable/legume and meat/fish intake (%EI), and individual characteristics.

				Bandung		Sumedang	
*Grain/tubers intake (%EI)*							
	Categorical variables			adjusted mean	p-value	adjusted mean	p-value
		Sex			0.37		0.92
			Male	54.5		65.1	
			Female	57.3		64.9	
		Occupation			0.01		0.10
			Housewife	56.5		66.1	
			Non-sedentary	61.0		65.3	
			Sedentary	63.4		65.6	
			Student	53.9		68.7	
			Other	44.7		59.3	
	Continuous variables			coefficient	p-value	coefficient	p-value
		Age		-0.03	0.75	0.30	<0.01
		Per capita income*		0.56	0.89	0.23	0.93
	Dummy variable				p-value		p-value
		Household			<0.01		<0.01
*Vegetables/legumes intake (%EI)*							
	Categorical variables			adjusted mean	p-value	adjusted mean	p-value
		Sex			0.04		0.56
			Male	15.2		8.4	
			Female	10.2		7.7	
		Occupation			0.12		0.23
			Housewife	18.9		7.8	
			Non-sedentary	14.6		6.7	
			Sedentary	8.9		10.7	
			Student	11.3		7.9	
			Other	9.7		7.2	
	Continuous variables			coefficient	p-value	coefficient	p-value
		Age		0.09	0.24	0.00	0.93
		Per capita income*		-4.79	0.11	-0.40	0.76
	Dummy variable				p-value		p-value
		Household			<0.01		<0.01
*Meat/fish intake (%EI)*							
	Categorical variables			adjusted mean	p-value	adjusted mean	p-value
		Sex			0.97		0.17
			Male	9.6		6.1	
			Female	9.5		8.0	
		Occupation			0.71		0.97
			Housewife	8.3		7.9	
			Non-sedentary	8.9		6.9	
			Sedentary	7.9		7.2	
			Student	10.7		6.7	
			Other	12.0		6.5	
	Continuous variables			coefficient	p-value	coefficient	p-value
		Age		-0.01	0.84	-0.05	0.25
		Per capita income*		2.08	0.35	0.65	0.66
	Dummy variable				p-value		p-value
		Household			<0.01		0.24

*million rupiah per month. EI: energy intake (kcal).

Tables 4 and 5 show the results from additional analyses that incorporated education and BMI variables, and were limited to adult samples. BMI was negatively associated with the total energy intake after adjusting for the basal metabolic rate in Sumedang, although the effect was small. Similarly, educational background was associated with energy intake from vegetable/legumes intake in Sumedang. A significant difference was found between the primary or lower education category and the junior high school category.

**Table 4:** Association between energy intake pattern and individual characteristics including education and BMI (adult samples only).

				Bandung		Sumedang	
*Energy intake (EI/BMR)*							
	Categorical variables			adjusted mean	p-value	adjusted mean	p-value
		Sex			0.25		0.64
			Male	1.20		1.22	
			Female	1.44		1.25	
		Education			0.84		0.51
			Higher	1.35		1.16	
			Junior high school	1.37		1.28	
			Primary or lower	1.24		1.26	
		Occupation			0.60		0.24
			Housewife	1.22		1.26	
			Non-sedentary	1.40		1.32	
			Sedentary	1.14		1.20	
			Student	1.09		-	
			Other	1.76		1.16	
	Continuous variables			coefficient	p-value	coefficient	p-value
		Age		0.00	0.73	0.00	0.66
		Body mass index		-0.01	0.54	-0.02	0.01
		Per capita income*		0.14	0.41	0.09	0.18
	Dummy variable				p-value		p-value
		Household			0.22		<0.01
*Protein intake (%EI)*							
	Categorical variables			adjusted mean	p-value	adjusted mean	p-value
		Sex			0.58		0.12
			Male	11.6		8.9	
			Female	12.3		9.8	
		Education			0.72		0.51
			Higher	12.0		9.6	
			Junior high school	12.4		8.8	
			Primary or lower	11.4		9.6	
		Occupation			0.77		0.81
			Housewife	11.9		9.0	
			Non-sedentary	11.9		9.5	
			Sedentary	13.1		9.3	
			Student	10.7		-	
			Other	12.1		9.7	
	Continuous variables			coefficient	p-value	coefficient	p-value
		Age		-0.01	0.84	-0.03	0.17
		Body mass index		-0.04	0.68	-0.08	0.21
		Per capita income*		0.03	0.97	-0.34	0.50
	Dummy variable				p-value		p-value
		Household			0.26		0.01
*Carbohydrate intake (%EI)*							
	Categorical variables			adjusted mean	p-value	adjusted mean	p-value
		Sex			0.32		0.36
			Male	63.4		71.3	
			Female	59.8		69.7	
		Education			0.64		0.38
			Higher	61.5		69.4	
			Junior high school	59.8		72.7	
			Primary or lower	63.5		69.5	
		Occupation			0.18		0.64
			Housewife	63.4		71.1	
			Non-sedentary	56.6		71.1	
			Sedentary	62.0		71.6	
			Student	71.3		-	
			Other	54.8		68.3	
	Continuous variables			coefficient	p-value	coefficient	p-value
		Age		0.13	0.32	0.12	0.13
		Body mass index		-0.43	0.15	0.19	0.35
		Per capita income*		1.95	0.51	-0.11	0.95
	Dummy variable				p-value		p-value
		Household			0.13		<0.01
*Fat intake (%EI)*							
	Categorical variables			adjusted mean	p-value	adjusted mean	p-value
		Sex			0.33		0.30
			Male	23.4		17.6	
			Female	27.1		19.0	
		Education			0.80		0.41
			Higher	25.2		19.2	
			Junior high school	26.6		16.6	
			Primary or lower	24.0		19.1	
		Occupation			0.18		0.54
			Housewife	23.5		18.1	
			Non-sedentary	30.4		17.8	
			Sedentary	23.0		17.2	
			Student	15.5		-	
			Other	33.9		20.3	
	Continuous variables			coefficient	p-value	coefficient	p-value
		Age		-0.15	0.26	-0.10	0.14
		Body mass index		0.43	0.17	-0.15	0.36
		Per capita income*		-1.17	0.70	0.25	0.85
	Dummy variable				p-value		p-value
		Household			0.11		<0.01

*million rupiah per month. EI: energy intake (kcal). BMR: basal metabolic rate (kcal).

**Table 5:** Association between grain/tuber, vegetable/legume and meat/fish intake (%EI), and individual characteristics including education and BMI (adult samples only).

				Bandung		Sumedang	
*Grain/tubers intake (%EI)*							
	Categorical variables			adjusted mean	p-value	adjusted mean	p-value
		Sex			0.94		0.71
			Male	56.3		69.9	
			Female	55.9		68.8	
		Education			0.39		0.06
			Higher	53.0		66.6	
			Junior high school	53.9		75.9	
			Primary or lower	61.6		65.6	
		Occupation			0.41		0.85
			Housewife	60.2		69.5	
			Non-sedentary	61.2		70.3	
			Sedentary	62.4		70.8	
			Student	52.0		-	
			Other	44.8		66.9	
	Continuous variables			coefficient	p-value	coefficient	p-value
		Age		-0.34	0.11	0.36	0.01
		Body mass index		0.27	0.57	0.52	0.15
		Per capita income*		0.00	0.94	-0.35	0.90
	Dummy variable				p-value		p-value
		Household			0.07		<0.01
*Vegetables/legumes intake (%EI)*							
	Categorical variables			adjusted mean	p-value	adjusted mean	p-value
		Sex			0.33		0.43
			Male	18.4		8.1	
			Female	13.4		6.9	
		Education			0.63		0.02
			Higher	18.0		7.8	
			Junior high school	17.2		4.1	
			Primary or lower	12.6		10.6	
		Occupation			0.49		0.08
			Housewife	18.4		6.9	
			Non-sedentary	14.2		4.9	
			Sedentary	13.5		8.6	
			Student	26.3			
			Other	7.2		9.5	
	Continuous variables			coefficient	p-value	coefficient	p-value
		Age		0.28	0.14	-0.12	0.09
		Body mass index		-0.18	0.68	-0.25	0.18
		Per capita income*		0.00	0.14	-0.77	0.59
	Dummy variable				p-value		p-value
		Household			0.28		<0.01
*Meat/fish intake (%EI)*							
	Categorical variables			adjusted mean	p-value	adjusted mean	p-value
		Sex			0.96		0.42
			Male	11.6		5.6	
			Female	11.4		7.0	
		Education			0.43		0.68
			Higher	9.3		7.0	
			Junior high school	12.1		4.9	
			Primary or lower	13.1		7.1	
		Occupation			0.05		0.90
			Housewife	9.6		7.5	
			Non-sedentary	8.8		6.0	
			Sedentary	9.9		6.0	
			Student	6.3		-	
			Other	22.8		5.9	
	Continuous variables			coefficient	p-value	coefficient	p-value
		Age		-0.16	0.10	-0.12	0.15
		Body mass index		0.19	0.39	-0.15	0.46
		Per capita income*		0.00	0.28	0.71	0.66
	Dummy variable				p-value		p-value
		Household			0.04		0.23

*million rupiah per month. EI: energy intake (kcal).

*Post-hoc* power analyses showed that the statistical power in the associations between individual characteristics and dietary/energy intake pattern indicators varied from 0.05 to 0.85 (mean = 0.19, SD = 0.16) for Bandung and 0.05 to 0.97 (mean = 0.28, SD = 0.27) for Sumedang. Although the sample size was considerably smaller in Bandung (n = 85) than in Sumedang (n = 201), the power was not necessarily lower in Bandung.

## Discussion

Focusing on urban-rural differences, this study explored the associations between dietary/energy intake patterns and individual characteristics in Bandung (an urban area) and Sumedang (a rural area). In Sumedang, five out of seven dietary/energy intake pattern indicators (total energy intake and energy intake from carbohydrates, fat, grains/tubers, and vegetables/legumes) were significantly associated with individual characteristics, such as age, occupation, education level, and BMI. In comparison, in Bandung, only two indicators (energy intake from grain/tubers and vegetables/legumes) were related to occupation or sex, and neither total energy intake nor energy intake from protein, carbohydrates, or fat was associated with any of the assessed factors.

Older age was associated with a higher energy intake from carbohydrates, but only in Sumedang. A similar trend was observed with respect to the association between age and energy intake from grain/tubers. These trends were likely due to rice and sweet potatoes being the least expensive sources of energy in Sumedang; therefore, the elderly maintain their customary levels of consumption of these food items to satisfy their hunger. Conversely, young people derived more energy from fat. The main sources of fat were fried tempeh, fried eggs, and fried chicken, which comprised 26% of the total fat intake of the whole subjects in Sumedang. Unlike rice and sweet potatoes, which are often grown in their own fields, people usually have to purchase the ingredients required to cook such fatty food items. Consequently, fat can be considered a luxury nutrient. Previous studies on intra-household food allocation reported that younger children were favored in some settings [26, 27], while elderly people were favored in other settings. This effect has been attributed to the differential valuation of household members [28].

Similarly, sedentary and non-sedentary workers in Sumedang, *i*.*e*., those who worked outside the home, showed the same trends in carbohydrate and fat intakes as elderly people; they derived more energy from carbohydrates and less from fat. It is reasonable to infer that, regardless of the type of job, working outside requires more energy, and people with such vocations thus consume more carbohydrates due to economic considerations. This might be underlie the association seen between occupation and grain/tuber intake observed in the Bandung cohort.

Among Bandung cohort, multivariate analysis showed that males obtained approximately 50% more energy from vegetable/legume intake than did females. However, this seemed to be offset by the high vegetable/legume intake among housewives. Indeed, the median energy intake from vegetables/legumes was higher in females (Fig 1), and there was no significant difference by sex in the bivariate analysis (Kruskal–Wallis equality-of-population test, *p* = 0.64).

Education level was associated with vegetable/legume intake among adults in Sumedang. However, the association was not linear; those who were in the lowest education category had the highest vegetable/legume intake, and those in the intermediate category the lowest. Although this finding might relate to differences in the level of knowledge of nutrition and eating habits acquired during adolescence, more detailed study is required to fully explain this finding.

In addition to the individual relationships between participant characteristics and dietary/energy intake patterns, it is important to note the overall contrast found between rural and urban areas. Many of the associations between dietary/energy intake and individual characteristics found in Sumedang were absent in Bandung. These results are consistent with those of previous studies. Using simulations, Drewnowski and Popkin [29] showed an interaction between urbanization and gross national product per capita in their effects on diet, which indicated that the decrease in energy intake from carbohydrates associated with income was smaller in a highly urbanized area than in a less urbanized area. Consequently, the differences found between the urban and rural areas in this study should not be attributed solely to the different sample sizes.

One possible explanation for the lack of an association between food intake patterns and individual characteristics in urban areas is that the food items affordable only to those of higher economic status in rural areas are prevalent among all socioeconomic groups in urban areas. Therefore, differences in socioeconomic status were not apparent. This effect has also been proposed to explain the differences in the relationship between socioeconomic status and energy intake by country [5]. Similar explanations have been posited in the context of the global trends in fat consumption, which became less dependent on gross domestic product during the three decades prior to 1990 [29]. It has been suggested that this is due to the weaker association between animal fat and income and the greater availability of vegetable fat, independent of income. The association between income and sweetener consumption may also have weakened with increased urbanization.

Another possible explanation is related to food availability, accessibility, and variety. Our overall results indicate that the dietary/energy intake patterns of individuals in Sumedang are determined more strongly by socioeconomic status, while those of individuals in Bandung are affected more by other factors, such as personal preferences or circumstances for example. Indeed, the variation in available food outlets is much greater in urban than in rural areas. In the survey areas of Sumedang, people usually buy fresh foods from vendors, which limits their choices according to their budget. There are only a few restaurants in daily living areas, most of which serve classic Indonesian dishes. In contrast, in Bandung the fresh food markets offer a greater variety of choices for each product type. Restaurants are also more widespread and offer a wider range of cuisines from around the world. This greater food availability, accessibility, and variety may cause individual preferences to become more diverse, even among those who share the same characteristics, and such preferences may be reflected in food choices. Consequently, individual socioeconomic status may not be related to food intake.

Moreover, when there are a wider variety of shops, there is likely to be a wider price range, which could explain the lack of an association between dietary/energy intake and socioeconomic status. Food items that are difficult to afford in Sumedang may be available at cheaper prices in Bandung. As a consequence, in Bandung most people may be able to afford their preferred foods, in contrast to Sumedang.

It has been said that during nutrition transitions, individuals with higher socioeconomic status shift their diets to consume less carbohydrates and rice and more protein, fat, and meat [30]. This principle is consistent with our results. The association between dietary intake and socioeconomic status observed in Sumedang suggests that it is in the middle of a nutrition transition. On the other hand, Bandung, which is considered more developed and urbanized, seems to be past this phase. Indeed, shifts in dietary patterns are thought to occur in urbanized areas first [29]. A gradient in the nutrition transition can exist even within a single region, and as the transition continues, the relationship between individual characteristics and food intake patterns changes.

Even though this case study focused on West Java, the observed urban-rural difference in the relationship between individual characteristics and dietary/energy intake suggests the need for health programs to be area-specific, since populations at risk of diet-related chronic diseases may differ among geographical areas. For example, according to our results, nutrition education would be more effective if it were tailored for different age groups, since there were significant differences in energy/dietary intake patterns among age groups. Therefore, to plan effective measures for disease prevention, a thorough understanding of typical intake patterns in the affected area is necessary.

### Strengths and limitations

This study had several limitations. Owing to the selective sampling methods used, the study population may not have been fully representative of the urban and rural Sundanese populations. This decreases the generalizability of the results to the Sundanese population as a whole. Moreover, there was a disparity in sample size between the two areas evaluated. The small number of samples in Bandung might have been insufficient to capture the diverse characteristics of the subjects, especially in terms of food intake patterns, which could have introduced a bias. The limited number of samples also prevented further analysis involving detailed categorization of food items. Another limitation is the possible bias introduced by weighed food records. As reported previously [31], the possibility of underreporting dietary intake among overnourished individuals cannot be excluded. Seasonal effects should be mentioned as well: the field surveys in both areas were conducted during the dry season, and the participants’ dietary patterns during the rainy season were not assessed. Nevertheless, results yielded from systematic field surveys improve our understanding of eating behavior and its predictors, especially in combination with other case studies.

This study also has many strengths. In Indonesia, including West Java, detailed surveys of diets have been limited to children and pregnant women due to greater interest in undernutrition in these groups. By surveying the entire population, i.e., by including subjects regardless of age or gender, this study obtained more informative data. Another strength was the use of weighed food records. This method is subject to fewer biases including recall bias, which is common with other dietary survey methods. In addition, by conducting surveys in two Sundanese-predominant areas with different levels of development, it was possible to compare food intake patterns and their determinants according to urbanization as a focal variable.

## Conclusion

In conclusion, the results of this study suggest that the relationships between demographic/socioeconomic characteristics and dietary/energy intake patterns differ between rural and urban areas in West Java, Indonesia. In rural areas, age, sex, education, and occupation were related to dietary/energy intake patterns, while few characteristics were associated with energy intake in urban areas. While the scope of the study was limited, these results suggest that different strategies are needed for rural and urban areas to develop strategies to aid populations at risk of diet-related diseases.

## Supporting information

S1 Table Dietary/energy intake patterns by area, adult/child status, and sex. (XLSX)
